# Child‐level double burden of malnutrition in the MENA and LAC regions: Prevalence and social determinants

**DOI:** 10.1111/mcn.12923

**Published:** 2019-12-11

**Authors:** Hala Ghattas, Yubraj Acharya, Zeina Jamaluddine, Moubadda Assi, Khalil El Asmar, Andrew D. Jones

**Affiliations:** ^1^ Faculty of Health SciencesAmerican University of Beirut Beirut Lebanon; ^2^ Department of Health Policy and Administration The Pennsylvania State University Pennsylvania; ^3^ Department of Nutritional Sciences, School of Public Health University of Michigan Ann Arbor Michigan

**Keywords:** children, double burden, LAC, MENA, overweight, stunting

## Abstract

Although the prevalence of obesity has rapidly increased in the low‐ and middle‐income countries of the Middle East and North Africa (MENA) and Latin America and the Caribbean (LAC) regions, child undernutrition remains a public‐health challenge. We examined region‐specific sociodemographic determinants of this double burden of malnutrition, specifically, the co‐occurrence of child stunting and overweight, using Demographic and Health Survey and Multiple Indicator Cluster Survey data (2003–2016) from 11 countries in the MENA (*n* = 118,585) and 13 countries in the LAC (*n* = 77,824) regions. We used multiple logistic regressions to model region‐specific associations of maternal education and household wealth with child nutritional outcomes (6–59 months). The prevalence of stunting, overweight, and their co‐occurrence was 24%, 10%, and 4.3% in children in the MENA region, respectively, and 19%, 5%, and 0.5% in children in the LAC region, respectively. In both regions, higher maternal education and household wealth were significantly associated with lower odds of stunting and higher odds of overweight. As compared with the poorest wealth quintiles, decreased odds of co‐occurring stunting and overweight were observed among children from the second, third, and fourth wealth quintiles in the LAC region. In the MENA region, this association was only statistically significant for the second wealth quintile. In both regions, double burden was not statistically significantly associated with maternal education. The social patterning of co‐occurring stunting and overweight in children varied across the two regions, indicating potential differences in the underlying aetiology of the double burden across regions and stages of the nutrition transition.

**Key messages**

- In the MENA region, the prevalence of stunted–overweight children was higher than expected.
- Wealth drives the co‐occurrence of stunting and overweight among children in different directions in the MENA and LAC regions.
- Targeting population nutritional intervention programs to both stunting and overweight is a challenge, and it will be important to account for the possible coexistence of undernutrition and overnutrition in the same child.

## 1 BACKGROUND

A global nutrition transition is under way, characterized by a rapid shift in diet composition and behavioural trends towards increased intakes of foods high in energy in the form of saturated fats and sugars and decreased intakes of nutrient‐ and fibre‐rich foods (B. M. Popkin, 2004). This transition has translated into a rise in overweight across all age groups globally (Abarca‐Gómez et al., 2017). Overweight and obesity prevalence are particularly high in the Middle East and North Africa (MENA) and Latin America and the Caribbean (LAC) regions, whereas stunting remains a public health challenge in subpopulations in these regions (Black et al., 2013; Farrag, Cheskin, & Farag, 2017; Ng et al., 2014; Rivera, Pedraza, Martorell, & Gil, 2014).

The co‐occurrence of undernutrition and overnutrition within populations, households, and even individuals has been described in these contexts and termed as the double burden of malnutrition (B. M. Popkin, 2004). Population‐level double burden refers to the prevalence of undernutrition and overweight in the same country or community. Whereas household‐level double burden, has been defined as the coexistence of undernutrition in children and overweight in adults within the same household (Black et al., 2013; Gubert, Spaniol, Segall‐Corrêa, & Pérez‐Escamilla, 2017; Jones, Acharya, & Galway, 2016; Wojcicki, 2014). Both population‐level and household level double burdens have been relatively well documented in the LAC region, with findings highlighting that stunting, anaemia and overweight are prevalent in women and children in these countries (Atalah, Amigo, & Bustos, 2014; Conde & Monteiro, 2014; Freire, Silva‐Jaramillo, Ramírez‐Luzuriaga, Belmont, & Waters, 2014; Gubert et al., 2017; Kroker‐Lobos, Pedroza‐Tobías, Pedraza, & Rivera, 2014; Ramirez‐Zea, Kroker‐Lobos, Close‐Fernandez, & Kanter, 2014; Rivera et al., 2014; Sarmiento et al., 2014; Severi & Moratorio, 2014). The rapid increases in overweight and obesity in the LAC region have been paralleled by similar surges in the MENA region (Abarca‐Gómez et al., 2017; Ng et al., 2014). Although one analysis found height‐for‐age *z* score (HAZ) to be correlated with body mass index‐for‐age *z* score among children under the age of 5 (El Taguri et al., 2009), few studies have described double burdens in the MENA region.

Much of the literature has described the household‐level double burden of maternal overweight and childhood stunting (El Kishawi, Soo, Abed, & Muda, 2016; Garrett & Ruel, 2003; Kosaka & Umezaki, 2017). Factors associated with this double‐burden include (a) urbanicity, with higher odds of double‐burden in urban and peri‐urban areas as compared with rural areas (Jones et al., 2016); (b) economic status (income and wealth), with some positive associations found between higher income and double burden although some studies show no association (Bassett, Romaguera, Giménez, Lobo, & Samman, 2014; Kosaka & Umezaki, 2017; Saibul et al., 2009); and (c) maternal or head of household education, where the associations are mixed (Kosaka & Umezaki, 2017).

Few studies have thus far focused on individual‐level double burdens such as overweight and anaemia coinciding in the same individual (women or children; Eckhardt, Torheim, Monterrubio, Barquera, & Ruel, 2008; Gartner et al., 2014; Pinhas‐Hamiel et al., 2003) and the coexistence of stunting and overweight in individual children (Bates, Gjonca, & Leone, 2017; Fernald & Neufeld, 2007; Mamabolo, Alberts, Steyn, Delemarre‐van de Waal, & Levitt, 2005; Barry M Popkin, Richards, & Montiero, 1996; Said‐Mohamed, Bernard, Ndzana, & Pasquet, 2012; Varela‐Silva et al., 2012; Wang et al., 2009).

Bates et al. (2017) recently reported prevalences of stunted–overweight in under‐5‐year‐old children from 79 low‐ and middle‐income countries and estimated that approximately 10 million children are concurrently stunted and overweight in these countries (Bates et al., 2017). The authors argue that this is a neglected phenomenon that requires further attention and that more research into the determinants of co‐occurring stunting and overweight is needed (Bates et al., 2017).

Household wealth and maternal education are well‐documented key determinants of childhood nutritional status: both stunting and overweight (Black et al., 2013; Hong & Mishra, 2006; Keino, Plasqui, Ettyang, & van den Borne, 2014; Vollmer et al., 2014). Lower wealth and maternal education are both associated with higher prevalence of stunting, whereas the associations of these factors with childhood overweight are not consistent (Keino et al., 2014; Kosaka & Umezaki, 2017; Tzioumis, Kay, Bentley, & Adair, 2016). Differentials exist by level of economic development and stage of the nutrition transition; in low‐income countries, most studies point to positive associations between household wealth and maternal education and childhood overweight, whereas in high‐income countries, overweight appears to be more common in low socioeconomic and education groups, with mixed results in middle‐income countries that have faced rapid changes in food environments and parallel increases in the prevalence of overweight (Martorell, Khan, Hughes, & Grummer‐Strawn, 2000; Subramanian, Perkins, Ozaltin, & Davey Smith, 2011). Some studies also suggest that there may be interactions between wealth and education in overweight differentials (Aitsi‐Selmi et al., 2012). Household wealth and maternal education are thus likely to also explain variability in the double burden of stunting and overweight in children, yet few studies have explored these (Fernald & Neufeld, 2007).

The aim of the present analysis is to describe the double burden of stunting and overweight in under‐5‐year‐old children at both the population and the individual level in low‐ and middle‐income countries of the MENA and LAC regions. We also examine the associations between household wealth and maternal education and stunting, overweight, and their co‐occurrence in children across the two regions.

## 2 METHODS

### 2.1 Data sources

Publicly available data from the multiple indicator cluster surveys (MICS available‐ http://mics.unicef.org/ Accessed 2018 June 1) and the demographic and health surveys (DHS available‐ https://dhsprogram.com/Data/. Accessed 2018 June 1) were used. Both MICS and DHS are household survey initiatives that collect comparable, nationally representative data from multiple countries including demographic, economic, health, and nutrition data for women and children. The standardized designs of these surveys allow for cross‐national comparisons of indicators. Both MICS and DHS surveys use a multistage stratified cluster sampling design where each primary unit has a defined probability of selection.

### 2.2 Sample definition

We included the most recent publicly available data sets from countries of the MENA and LAC regions that collected anthropometric data for under‐5‐year‐old children (length/height, weight), and household data on assets for the construction of a wealth index. Eight MICS and four DHS data sets from the MENA region and seven MICS and six DHS data sets from the LAC region were available and met inclusion criteria. We used the anthropometry module from the questionnaire for under‐5‐year‐old children and the education and household characteristics modules from the household questionnaire. Data on child anthropometric variables, age, gender, maternal education, household assets, and urbanicity were extracted and coded to harmonize categorization of variables across data sets. The data sets from each region were independently pooled into 2 regional (MENA and LAC) data sets to produce of region‐specific estimates with high precision. We excluded all infants under the age of 6 months from this analysis. Table 1 lists the data sets used and sample size of the data sets across the different countries for 6–59‐month‐old children.

**Table 1:** Data sets included from the Middle East and North Africa and Latin American and Caribbean regions

MENA country, year (source)	Sample size	LAC country, year (source)	Sample size
Algeria, 2013 (MICS)	13,077	Barbados, 2012 (MICS)	430
Djibouti, 2006 (MICS)	2,073	Belize, 2015 (MICS)	2,372
Egypt, 2014 (DHS)	13,857	Colombia, 2010 (DHS)	15,402
Iraq, 2011 (MICS)	32,425	Dominican Republic, 2013 (DHS)	3,076
Jordan, 2012 (DHS)	6,071	El Salvador, 2014 (MICS)	6,825
Morocco, 2003 (DHS)	5,309	Guatemala, 2015 (DHS)	10,774
Palestine, 2014 (MICS)	7,151	Guyana, 2014 (MICS)	3,068
Sudan, 2014 (MICS)	12,538	Haiti, 2012 (DHS)	3,599
Syria, 2006 (MICS)	9,854	Honduras, 2011 (DHS)	9,154
Tunisia, 2012 (MICS)	2,593	Mexico, 2015 (MICS)	7,400
Yemen, 2013 (DHS)	13,637	Paraguay, 2016 (MICS)	4,231
		Peru, 2012 (DHS)	8,489
		Suriname, 2010 (MICS)	3,004
Pooled MENA	118,585	Pooled LAC	77,824

Abbreviations: DHS, demographic and health surveys; LAC, Latin American and Caribbean regions; MENA, Middle East and North Africa; MICS, Multiple Indicator Cluster Surveys.

### 2.3 Measures

#### 2.3.1 Dependent variables

To analyse the nutritional status of children aged 6–59 months, indicators for stunting and overweight were derived according to World Health Organization Child Growth standards (World Health Organization, 2015); *z* scores for HAZ and weight‐for‐height were calculated using the Stata command *zscore06*. Stunting was defined as HAZ < 2 *SD* below the reference median value and overweight as weight‐for‐height > 2 *SD* above the median. Outliers were defined as any index value with a *z*‐score > 6 *SD* or < 6 *SD* away from the median and were therefore excluded from the analysis. Child‐level double burden was defined as the co‐occurrence of stunting and overweight in a single individual, according to the above classification assuming these conditions are independent from one another. If any of the measurements used to calculate the standardized *z* scores were missing, the outcome variable was not generated for that particular individual.

#### 2.3.2 Independent variables

The independent variables of interest were maternal education and wealth, the primary social determinants of child malnutrition. Maternal education was standardized across all data sets as a categorical variable (no education/primary education/secondary education or more). Wealth index is a measure of household's cumulative living standards and is calculated using principal component analysis by DHS and MICS. The standard wealth index is based on household ownership of durable assets (such as a television and a refrigerator), materials used for housing construction (such as the type of floor), and variables such as household's main source of drinking water and sanitation facilities (Filmer & Pritchett, 2001; Rutstein & Johnson, 2004). We recalculated the wealth index by excluding hygiene and sanitation variables, in order to explore the individual associations between these variables and nutritional outcomes. The resulting index captures the ownership of assets (viz, radio, television, refrigerator, bicycle, motorcycle, and car), type of flooring, and access to electricity. This set of factors reflects information that was uniformly collected in all countries. The first principal component was used to predict a unidimensional index, which was then categorized into five quintiles.

Hygiene was represented by a dichotomous indicator measuring the source of water (piped or otherwise), whereas sanitation was represented by a dichotomous indicator measuring the type of toilet (piped sewage/septic tank or otherwise). A dichotomous variable was created for urban–rural dwelling. Child age was categorized into 3 categories (age 6–11, 12–23, and 24–59 months) and treated as a discrete variable.

### 2.4 Statistical analyses

Analyses were conducted on Stata 13.0 (StataCorp, College Station, TX, USA). The prevalence of single and double burdens was estimated for each country. A total prevalence estimate (unweighted by country size) was also generated for each region. We calculated the expected prevalence of double burden within each data set by multiplying the prevalence of overweight by the prevalence of stunting in children. The difference between observed and expected prevalences of the double burden was calculated for each country.

Regional pooled data sets were then used to examine the correlates of stunting, overweight, and child‐level double burden. Bivariate analyses were used to describe the outcomes of interest across the selected social determinants, and the *χ* ^*2*^ test was used to detect statistically significant associations between wealth or education and nutritional status. Bivariate logistic regression models were used to calculate unadjusted odds ratios of the association between the various sociodemographic determinants and each of the three outcomes: child stunting, overweight, and double burden of stunting and overweight. Multivariable logistic regression models of the correlates of stunting, overweight, and child‐level double burden included variables with a strong theoretical basis: wealth, maternal education, hygiene, sanitation, urbanicity, age, and gender. All models were adjusted for country fixed effects and within country clustering effect.

Interactions between maternal education and wealth index were tested, and data were stratified by maternal education level to further examine significant interactions between maternal education and wealth (Aitsi‐Selmi, Bell, Shipley, & Marmot, 2014). All country‐level estimates presented were weighted using in‐country sampling weights as provided by MICS and DHS. A *p* value of.05 was used to indicate statistical significance. All data were publicly available; therefore, ethical review was not required.

## 3 RESULTS

The analytical sample included 118,585 children aged 6–59 months in the MENA region and 77,824 children in the LAC region (Table 1).

Table 2 presents the prevalence of stunting, overweight and co‐occurring stunting, and overweight among 6–59‐month‐old children in both regions, alongside GDP per capita; Table S1 presents the prevalence of maternal education levels by country. The prevalence of stunting in the MENA region varied from 7.4% in Palestine to 49.1% in Yemen and in the LAC region from 5.7% in Paraguay to 48.2% in Guatemala. Total (unweighted by country population size) regional prevalence estimates of stunting were slightly lower in the LAC region as compared with the MENA region. The prevalence of overweight in children in the MENA region was lowest in Yemen (2.1%) and highest in Syria (18.2%), whereas in the LAC region, it ranged from 3.2% in Guatemala and Haiti to 12.6% in Paraguay. Overall, total overweight prevalence was substantially lower in the LAC region (5.5%) as compared with the MENA region (10.0%). Consequently, the prevalence of the double burden was very low at 0.5% in the LAC region, whereas it was 4.3% in the MENA region. The prevalence of the double burden reached 1.9% in Barbados and 10.7% in Syria (numbers from Syria are from data collected in 2006 and predate the current ongoing conflict).

**Table 2:** Prevalence of stunting, overweight and stunted–overweight 6–59‐month‐old children in the MENA and LAC regions

MENA and LAC region		GDP per capita	Stunting (%)	Overweight (%)	Stunted–overweight double burden (Observed %)	Stunted–overweight double burden (Expected %)	Difference between observed and expected prevalence
MENA	Algeria, 2013	5,472	10.7	12.4	3.3	1.3	2.0
	Djibouti, 2006	966	33.5	13.9	7.4	4.6	2.7
	Egypt, 2014	3,328	22.4	15.4	8.0	3.4	4.5
	Iraq, 2011	5,855	20.4	10.9	4.8	2.2	2.6
	Jordan, 2012	4,088	7.6	3.9	0.4	0.3	0.1
	Morocco, 2003	1,722	23.8	13.0	5.6	3.1	2.5
	Palestine, 2014	2,961	7.4	7.6	1.0	0.6	0.4
	Sudan, 2014	2,177	41.2	2.6	1.7	1.1	0.6
	Syria, 2006	1,762	28.6	18.2	10.7	5.2	5.5
	Tunisia, 2012	4,138	8.7	14.1	2.5	1.2	1.3
	Yemen, 2013	1,580	49.1	2.1	1.2	1.0	0.1
	Pooled MENA	–	24.3	10.0	4.3	2.4	1.9
LAC	Barbados, 2012	16,536	6.4	11.1	1.9	0.7	1.2
	Belize, 2016	4,811	15.4	7.4	0.7	1.1	‐0.4
	Colombia, 2010	6,251	12.9	4.8	0.4	0.6	‐0.2
	Dominican Republic, 2013	6,027	6.7	7.1	0.6	0.5	0.2
	El Salvador, 2014	3,989	14.0	6.4	0.4	0.9	‐0.5
	Guatemala, 2015	3,924	48.2	3.2	0.7	1.6	‐0.8
	Guyana, 2014	4,031	10.7	5.3	1.0	0.6	0.4
	Haiti, 2012	767	22.2	3.2	0.9	0.7	0.2
	Honduras, 2011	2,121	23.5	5.0	0.5	1.2	‐0.7
	Mexico, 2015	9,143	13.1	5.0	0.3	0.7	‐0.4
	Paraguay, 2016	4,080	5.7	12.6	0.5	0.7	‐0.2
	Peru, 2012	6,388	18.4	7.0	0.3	1.3	‐1.0
	Suriname, 2010	8,303	7.6	3.8	0.5	0.3	0.2
	Pooled LAC	–	19.7	5.5	0.5	1.1	‐0.5

Abbreviations: LAC, Latin American and Caribbean regions; MENA, Middle East and North Africa.

The differences between observed and expected prevalence of stunted–overweight children ranged from 0.1% to 5.5% in the MENA region with a mean of 1.9%, whereas for the LAC region the difference ranged from −1.0% to 1.2% with a mean of −0.5% (Table 2). The observed prevalence of the double burden exceeded the expected prevalence in 5 of the 13 LAC countries and in all of the 11 MENA countries studied.

Table S2 presents bivariate analyses from aggregated LAC and MENA data sets showing associations between hypothesized correlates and stunting, overweight, and child‐level double burden.

**Figure 1:**
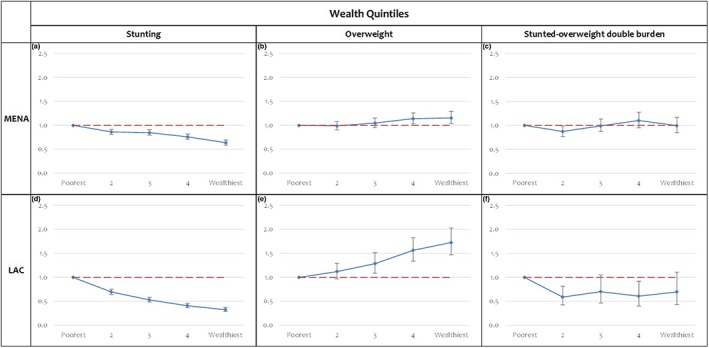
Odds of child stunting, overweight and the stunted–overweight double burden, by wealth quintiles in the MENA and LAC regions (OR; 95% CI). Multivariable regression models adjusted for child sex and age, maternal education, urbanicity, hygiene, country fixed effects, and within country clustering effect. LAC, Latin American and Caribbean regions; MENA, Middle East and North Africa

Multivariable models then assessed differentials in stunting, overweight, and co‐occurring stunting and overweight by wealth quintiles (Figure 1). Across both data sets, children in the lowest quintiles had the highest odds of being stunted. In the MENA region, children belonging to the richest subgroup had lower odds to be stunted as compared with children living in the poorest subgroup (0.63; 95% CI [0.58, 0.69]; Figure 1a). For the LAC region, the association was even stronger (0.32; 95% CI [0.28, 0.37]; Figure 1d).

Wealth inequalities were also observed in overweight. The correlation between overweight and wealth was more pronounced in the LAC region as compared with the MENA region. The richest subgroup in the MENA region was found to be 1.15 times more likely to be overweight as compared with the poorest subgroup (Figure 1b), whereas their LAC counterparts were 1.72 times more likely to be overweight as compared with the poorest subgroup (Figure 1e). Co‐occurring stunting and overweight among children was correlated with wealth in both regions. As compared with the poorest quintiles, lower odds of the stunted–overweight double burden were observed among children from the second, third, and fourth quintiles in the LAC region (Figure 1f). In the MENA region, this association was only statistically significant for the second wealth quintile (Figure 1c).

**Figure 2:**
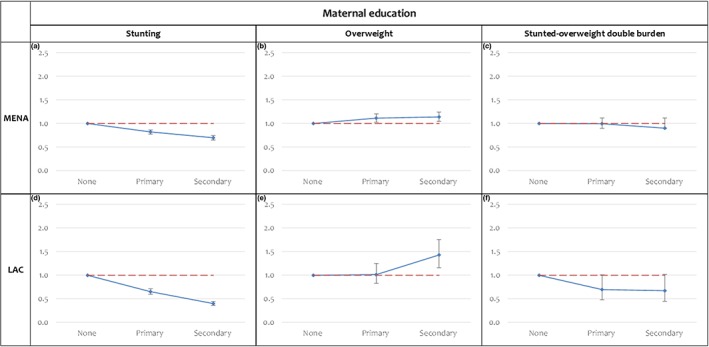
Odds of child stunting, overweight and the stunted–overweight double burden, by maternal education in the MENA and LAC regions (OR; 95% CI). Multivariable regression models adjusted for child sex and age, wealth quintiles, urbanicity, hygiene, country fixed effects, and within country clustering effect. LAC, Latin American and Caribbean regions; MENA, Middle East and North Africa

Differentials in child nutritional status were also investigated by maternal education for both regions (Figure 2). Across both regions, multivariable analysis showed that higher maternal education was associated with a statistically significantly lower odds of stunting (Figure 2a,d). This association was stronger in the LAC region (0.39; 95% CI [0.35, 0.44] for secondary education). The odds of child overweight increased with increased maternal education level in both regions. Secondary maternal education level was more strongly associated with child overweight in the LAC region (1.42; 95% CI [1.16, 1.75]; Figure 2e) than in the MENA region (1.14; 95% CI [1.04, 1.23]; Figure 2b). Maternal education was not statistically significantly associated with the co‐occurrence of stunting and overweight in either region (Figure 2c,f).

**Figure 3:**
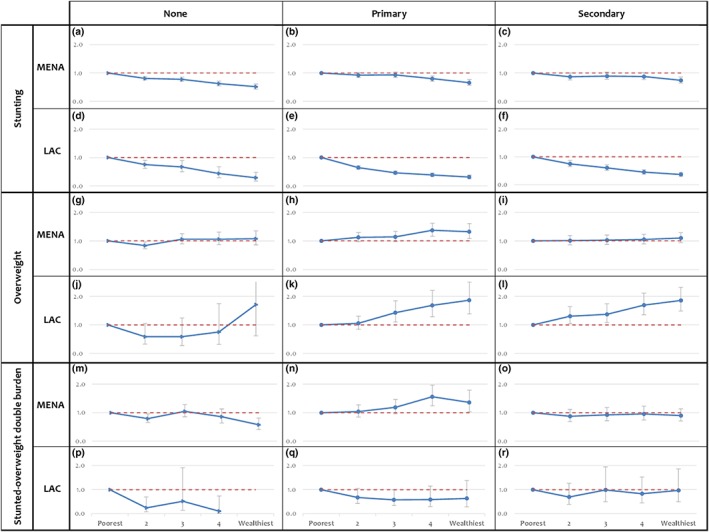
Odds of stunting, overweight and the stunted–overweight double burden, by wealth quintiles in children with mothers with varying educational status (none, primary, and secondary or more school attainment; OR; 95% CI). Multivariable regression models adjusted for child sex and age, urbanicity, hygiene, country fixed effects, and within country clustering effect. LAC, Latin American and Caribbean regions; MENA, Middle East and North Africa

To examine whether the association between wealth and child nutritional status outcomes varied according to maternal education, tests for interaction between wealth and maternal education were found to be significant for the MENA but not the LAC region. Data were stratified by maternal education level (none, primary, secondary plus) for both regions for comparative purposes. Within these strata, we examined the association of wealth with stunting, overweight, and the double burden while adjusting for sex, age, urbanicity, hygiene, and country effect (Figure 3). In the MENA region, the association between wealth and stunting was attenuated with increasing maternal education; in children of women with no education, odds of stunting were 0.5 (95% CI [0.4, 0.6]) in the richest compared with the poorest quintile, whereas in children of women with secondary education or higher, these odds were 0.7 (95% CI [0.6, 0.8]; Figure 3a–c); this was not the case in the LAC region (Figure 3e–f). Odds of overweight increased with increasing wealth quintiles among mothers educated to the primary level in both the MENA and LAC regions (Figure 3h,k). For mothers with higher education levels in the LAC region, the association between overweight and wealth followed a similar pattern (Figure 3j–l), whereas there was no association in the MENA region (Figure 3g–i).

In the MENA region, stunted–overweight children followed a similar pattern to overweight children (Figure 3m–o). However, the number of children with double burden in the LAC region was too small to allow us to explore these associations (Figure 3p–r).

## 4 DISCUSSION

We present here a comparative analysis of the associations of sociodemographic characteristics with the double burden of malnutrition across two global regions where the nutrition transition has been documented to be under way (Abarca‐Gómez et al., 2017). Whereas the literature generally describes prevalence estimates and studies burden associations within countries, this is the first paper to systematically compare regional patterns in child stunting, overweight, and the double burden and assess their determinants in the LAC and MENA regions.

Our findings confirm that in these regions, chronic undernutrition (stunting) is consistently rooted in poverty, with the odds of stunting significantly decreasing with increasing wealth and maternal education, consistent with global literature (Hong, 2007; Keino et al., 2014; D Makoka, 2013; Semba et al., 2008; Tzioumis et al., 2016; Vollmer et al., 2014). Our results also highlight the differential association of maternal education on stunting across the two regions. In the MENA region particularly, the effect of wealth on stunting is attenuated by maternal education (Figure 3), adding to the global evidence on the particular importance of maternal education for the prevention of child stunting in this region (Abuya, Ciera, & Kimani‐Murage, 2012; D. Makoka & Masibo, 2015; Negash, Whiting, Henry, Belachew, & Hailemariam, 2015; Semba et al., 2008; Wachs, Creed‐Kanashiro, Cueto, & Jacoby, 2005).

Although stunting is generally on the decline in these regions, obesity has steadily increased in adults and is increasingly reported among children (Abarca‐Gómez et al., 2017; Black et al., 2013; Ng et al., 2014). We find the prevalence of overweight among under‐5‐year‐old children to be higher in the MENA region as compared with LAC region. The findings also highlight stronger socioeconomic differentials in child overweight in the LAC region vis‐à‐vis the MENA region. This finding of generally high levels of overweight in the MENA region aligns with other evidence from the region highlighting elevated prevalence of obesity in children and women and may be explained by the particularly high penetration of globalized food markets in the MENA region and weaker policies to promote breastfeeding and regulate unhealthy foods, including to infants and children (Abarca‐Gómez et al., 2017; Akik, Ghattas, Filteau, & Knai, 2017; Colchero, Rivera‐Dommarco, Popkin, & Ng, 2017; Fraser, 2013). Additional cultural factors such as notions of beauty and health and social restrictions on physical activity may contribute to these differences across regions (Obermeyer, Bott, & Sassine, 2015; Sharara, Akik, Ghattas, & Obermeyer, 2018). It may be that the co‐occurrence of stunting and overweight, which was reported in LAC populations a decade ago, has stimulated some action at the policy level to reduce both these burdens (Hodge, Verstraeten, & Ochoa‐Avilés, 2016; Hoey & Pelletier, 2011; Huicho et al., 2016).

The prevalence of co‐occurring stunting and overweight among children is consequently low in the LAC region and may not have allowed sufficient statistical power for us to adequately investigate social determinants, whereas the numbers appear to represent a greater burden in the MENA region.

It has been argued that double burdens may not be an independent phenomenon, but rather a by‐product of the differences in pace at which chronic malnutrition is disappearing and overnutrition is appearing (Dieffenbach & Stein, 2012). These studies, focusing on the intrahousehold stunted child‐overweight mother double burden, have shown that in most countries the computed observed prevalence of stunted‐child, overweight‐mother pairs does not surpass the expected prevalence (Dieffenbach & Stein, 2012; Rivera et al., 2014). In the case of the present study, we observed a difference of around 2% between the expected and the observed prevalence of stunted–overweight children in the MENA region (Table 2), whereas the difference was close to 0 in the LAC region. These findings suggest that in the MENA region, the stunted–overweight child is a true phenomenon rather than a statistical artefact. Potential explanations for the existence of the stunted–overweight child include poor quality diets with limiting micronutrients, but sufficient caloric density (Fernald & Neufeld, 2007; Mamabolo et al., 2005; Barry M Popkin et al., 1996; Said‐Mohamed et al., 2012; Varela‐Silva et al., 2012; Wang et al., 2009), in‐utero epigenetic alterations (Barker, 2006; Kitsiou‐Tzeli & Tzetis, 2017), or poor early nutrition that alters the physiology of stunted children to accumulate fat instead of lean mass (Freire et al., 2014; Kroker‐Lobos et al., 2014; Ramirez‐Zea et al., 2014; Tzioumis et al., 2016).

Our data further suggest that the co‐occurrence of stunting and overweight among children is driven differentially in each region by the underlying burdens. In the LAC region, the direction of the associations of wealth and maternal education with the double burden follow those of stunting. Whereas in the MENA region, these associations follow those of overweight, even after stratifying for maternal education. This may reflect different etiologies underlying the double burden, the more advanced nutrition transition of the MENA region as compared with the LAC region or that these analyses are limited by the small samples of stunted–overweight children.

In the present analysis, children with the double burden were included in both the stunted only and the overweight only categories, although it has been suggested that the stunted–overweight double burden should be explored using mutually exclusive categories in order to better understand its etiology. However, when these categories were generated, the results remained unchanged for all the analyses presented (data not shown).

The advantages of using DHS and MICS data sets include the comparability across regions of surveys having used standardized methodologies, on representative samples. Our findings are limited, however, by the cross‐sectional nature of the data and our inability to examine temporal trends. Although we did not aim to capture changes in patterns and trends over time, it would be worth conducting such analysis on longitudinal data sets to better elucidate the effects of the nutrition transition on the stunted–overweight double burden. And though DHS and MICS are highly relied upon surveys for the estimation of child malnutrition globally, it is important to note that any systematic error that may have occurred in height measurement could exaggerate bias in the estimation of double burden prevalence. It should also be noted that although country‐level analyses utilized sampling weights provided by MICS and DHS, the pooled analyses do not adjust for population size of each country, as this would require country‐level data on population size for under‐5‐year‐old children. Another limitation of this analysis is the variability in the waves of MICS and DHS surveys included. Although we captured the most recent available surveys for both regions, for countries such as Syria and Yemen, these data predate ongoing conflicts and are unlikely to represent the current nutritional situation of children, which has been catastrophically affected (El Bcheraoui, Jumaan, Collison, Daoud, & Mokdad, 2018; Eshaq, Fothan, Jensen, Khan, & AlAmodi, 2017; Meiqari, Hoetjes, Baxter, & Lenglet, 2018).

## 5 CONCLUSION

The systematic comparison of stunting, overweight, and stunted–overweight burdens across two rapidly developing regions has highlighted that despite being on a similar development trajectory, nutritional burdens vary greatly and are driven differentially within and across the LAC and MENA regions. This highlights the need for regional or country‐level focus on these determinants, rather than global analyses, which may be divorced from local contexts and make decision‐making and specific policy formulation/recommendations more challenging.

The high prevalence of stunted–overweight children in the MENA region is of public health importance and requires further research on the pathways leading to this double burden, as well as its short‐ and long‐term impacts on health and development of these children. Policies and programs aiming to promote breastfeeding and regulate the marketing and sales of unhealthy foods to infants and children are needed, and there may be lessons to derive on this from the LAC region.

Targeting population nutritional intervention programs to both stunting and overweight is a challenge and should take into account the possible coexistence of undernutrition and overnutrition in the same household or individual. This is particularly relevant at a time when there have been shifts from in‐kind food assistance to cash‐based programs in many countries of these two regions.

## CONFLICTS OF INTEREST

Hala Ghattas, Yubraj Acharya, Zeina Jamaluddine, Moubadda Assi, Khalil El Asmar, and Andrew D. Jones have no conflicts of interest to declare.

## CONTRIBUTIONS

AJ and HG designed the study and developed the overall research plan; HG, AJ and KA oversaw the data analysis. YA, ZJ and MA analysed the data and performed statistical analysis; HG wrote the manuscript; AJ had the primary responsibility for final content. All authors (HG, YA, ZJ, MA, KA, AJ) have read and approved the final manuscript.

## Supporting information


**Table S1** Prevalence of maternal education level of 6‐59‐month‐old children in the MENA and LAC regions.
**Table S2** Unadjusted odds of stunting, overweight and child‐level double burden from aggregated LAC and MENA datasets.
