# Supplementary material for: Child‐level double burden of malnutrition in the MENA and LAC regions: Prevalence and social determinants
Source: Matern Child Nutr. 2019 Dec 11;16(2):e12923. doi: 10.1111/mcn.12923 (PMC7083402; doi:10.1111/mcn.12923)
Supplement: Supplementary file 1 — Table S1 Prevalence of maternal education level of 6‐59‐month‐old children in the MENA and LAC regions. Table S2 Unadjusted odds of stunting, overweight and child‐level double burden from aggregated LAC and MENA datasets. [file MCN-16-e12923-s001.docx]

**Supplementary**. Prevalence of maternal education level of 6-59-month-old children in the MENA and LAC regions.

|  |  | None (%) | Primary (%) | Secondary plus (%) |
| --- | --- | --- | --- | --- |
| **MENA** | Algeria, 2013 | 18.3 | 48.4 | 33.2 |
|  | Djibouti, 2006 | 62.6 | 18.9 | 18.3 |
|  | Egypt, 2014 | 17.9 | 8.6 | 73.4 |
|  | Iraq, 2011 | 17.9 | 50.0 | 32.0 |
|  | Jordan, 2012 | 2.1 | 6.4 | 91.3 |
|  | Morocco, 2003 | 63.1 | 17.6 | 19.2 |
|  | Palestine, 2014 | 0.4 | 30.6 | 68.9 |
|  | Sudan, 2014 | 42.6 | 34.7 | 22.7 |
|  | Syria, 2006 | 17.8 | 37.4 | 44.7 |
|  | Tunisia, 2012 | 12.8 | 33.6 | 53.5 |
|  | Yemen, 2013 | 55.7 | 31.8 | 12.3 |
|  | Pooled MENA | 25.3 | 33.7 | 41.0 |
| **LAC** | Barbados, 2012 | 0.0 | 1.71 | 98.2 |
|  | Belize, 2015 | 3.8 | 47.4 | 48.7 |
|  | Colombia, 2010 | 2.2 | 26.0 | 71.7 |
|  | Dominican Republic, 2013 | 2.4 | 33.8 | 63.7 |
|  | El Salvador, 2014 | 7.0 | 33.5 | 59.3 |
|  | Guatemala, 2015 | 19.0 | 52.9 | 27.9 |
|  | Guyana, 2014 | 2.0 | 14.4 | 83.5 |
|  | Haiti, 2012 | 21.3 | 43.0 | 35.6 |
|  | Honduras, 2011 | 4.8 | 59.3 | 35.7 |
|  | Mexico, 2015 | 2.0 | 17.3 | 80.5 |
|  | Paraguay, 2016 | 2.5 | 31.3 | 66.1 |
|  | Peru, 2012 | 3.1 | 28.4 | 68.4 |
|  | Suriname, 2010 | 14.0 | 30.0 | 55.9 |
|  | Pooled LAC | 7.4 | 35.9 | 56.7 |

**Supplementary 2-** Unadjusted odds of stunting, overweight and child-level double burden from aggregated LAC and MENA datasets.

|  |  | **MENA** | | | | **LAC** | | | |
| --- | --- | --- | --- | --- | --- | --- | --- | --- | --- |
|  |  | OR | 95%CI | | P value | OR | 95%CI | | P value |
| **Stunting** | **Sex (referent: male)** |  |  |  |  |  |  |  |  |
|  | female | 0.753 | 0.706 | 0.803 | <0.001 | 0.703 | 0.647 | 0.763 | <0.001 |
|  | **Age (referent: 12 to 23 months)** |  |  |  |  |  |  |  |  |
|  | 24 to 59 months | 1.711 | 1.587 | 1.845 | <0.001 | 1.650 | 1.502 | 1.814 | <0.001 |
|  | **Area (referent: urban)** |  |  |  |  |  |  |  |  |
|  | rural | 1.584 | 1.462 | 1.717 | <0.001 | 2.038 | 1.833 | 2.266 | <0.001 |
|  | **Maternal Education (referent: none)** |  |  |  |  |  |  |  |  |
|  | primary | 0.583 | 0.535 | 0.635 | <0.001 | 0.544 | 0.474 | 0.624 | <0.001 |
|  | secondary plus | 0.463 | 0.424 | 0.506 | <0.001 | 0.197 | 0.169 | 0.230 | <0.001 |
|  | **Wealth Quintiles (referent: poorest)** |  |  |  |  |  |  |  |  |
|  | 2 | 0.835 | 0.760 | 0.918 | <0.001 | 0.674 | 0.606 | 0.750 | <0.001 |
|  | 3 | 0.802 | 0.728 | 0.884 | <0.001 | 0.491 | 0.429 | 0.562 | <0.001 |
|  | 4 | 0.643 | 0.578 | 0.714 | <0.001 | 0.357 | 0.306 | 0.418 | <0.001 |
|  | wealthiest | 0.599 | 0.533 | 0.673 | <0.001 | 0.266 | 0.225 | 0.313 | <0.001 |
|  | **Water (referent: unimproved)** |  |  |  |  |  |  |  |  |
|  | water piped into dwelling or piped to plot/yard | 0.769 | 0.712 | 0.831 | <0.001 | 0.628 | 0.570 | 0.693 | <0.001 |
|  | **Sanitation (referent: unimproved)** |  |  |  |  |  |  |  |  |
|  | toilet has either piped sewage system or septic tank | 0.615 | 0.569 | 0.664 | <0.001 | 0.477 | 0.432 | 0.527 | <0.001 |
|  | **Currently breastfeeding (referent: no)** |  |  |  |  |  |  |  |  |
|  | yes | 1.173 | 1.093 | 1.258 | <0.001 | 1.575 | 1.426 | 1.740 | <0.001 |
| **Overweight** | **Sex (referent: male)** |  |  |  |  |  |  |  |  |
|  | female | 0.895 | 0.818 | 0.979 | 0.015 | 0.944 | 0.819 | 1.087 | 0.423 |
|  | **Age (referent: 12 to 23 months)** |  |  |  |  |  |  |  |  |
|  | 24 to 59 months | 1.118 | 1.020 | 1.226 | 0.017 | 0.752 | 0.654 | 0.864 | <0.001 |
|  | **Area (referent: urban)** |  |  |  |  |  |  |  |  |
|  | rural | 0.772 | 0.700 | 0.851 | <0.001 | 0.716 | 0.619 | 0.829 | <0.001 |
|  | **Maternal Education (referent: none)** |  |  |  |  |  |  |  |  |
|  | primary | 1.368 | 1.213 | 1.543 | <0.001 | 1.354 | 0.954 | 1.920 | 0.090 |
|  | secondary plus | 1.634 | 1.456 | 1.834 | <0.001 | 2.318 | 1.654 | 3.247 | <0.001 |
|  | **Wealth Quintiles (referent: poorest)** |  |  |  |  |  |  |  |  |
|  | 2 | 1.030 | 0.903 | 1.175 | 0.660 | 1.408 | 1.130 | 1.755 | 0.002 |
|  | 3 | 1.061 | 0.925 | 1.216 | 0.400 | 1.424 | 1.127 | 1.800 | 0.003 |
|  | 4 | 1.211 | 1.059 | 1.386 | 0.005 | 1.938 | 1.569 | 2.394 | <0.001 |
|  | wealthiest | 1.165 | 1.000 | 1.357 | 0.050 | 1.995 | 1.573 | 2.531 | <0.001 |
|  | **Water (referent: unimproved)** |  |  |  |  |  |  |  |  |
|  | water piped into dwelling or piped to plot/yard | 1.651 | 1.492 | 1.826 | <0.001 | 1.359 | 1.177 | 1.571 | <0.001 |
|  | **Sanitation (referent: unimproved)** |  |  |  |  |  |  |  |  |
|  | toilet has either piped sewage system or septic tank | 1.626 | 1.466 | 1.803 | <0.001 | 1.275 | 1.104 | 1.473 | 0.001 |
|  | **Currently breastfeeding (referent: no)** |  |  |  |  |  |  |  |  |
|  | yes | 0.757 | 0.693 | 0.828 | <0.001 | 0.935 | 0.799 | 1.094 | 0.403 |
| **Stunted-overweight double burden** | **Sex (referent: male)** |  |  |  |  |  |  |  |  |
|  | female | 0.849 | 0.746 | 0.967 | 0.013 | 1.004 | 0.651 | 1.548 | 0.986 |
|  | **Age (referent: 12 to 23 months)** |  |  |  |  |  |  |  |  |
|  | 24 to 59 months | 0.981 | 0.852 | 1.129 | 0.788 | 0.432 | 0.280 | 0.666 | <0.001 |
|  | **Area (referent: urban)** |  |  |  |  |  |  |  |  |
|  | rural | 1.007 | 0.871 | 1.165 | 0.920 | 1.125 | 0.734 | 1.726 | 0.589 |
|  | **Maternal Education (referent: none)** |  |  |  |  |  |  |  |  |
|  | primary | 0.976 | 0.814 | 1.170 | 0.792 | 0.990 | 0.465 | 2.108 | 0.978 |
|  | secondary plus | 1.211 | 1.029 | 1.426 | 0.021 | 0.864 | 0.401 | 1.861 | 0.708 |
|  | **Wealth Quintiles (referent: poorest)** |  |  |  |  |  |  |  |  |
|  | 2 | 0.875 | 0.725 | 1.056 | 0.164 | 0.780 | 0.465 | 1.307 | 0.345 |
|  | 3 | 0.897 | 0.736 | 1.093 | 0.280 | 0.813 | 0.403 | 1.639 | 0.562 |
|  | 4 | 0.914 | 0.747 | 1.119 | 0.385 | 0.610 | 0.323 | 1.155 | 0.129 |
|  | wealthiest | 0.877 | 0.705 | 1.090 | 0.236 | 0.775 | 0.377 | 1.593 | 0.488 |
|  | **Water (referent: unimproved)** |  |  |  |  |  |  |  |  |
|  | water piped into dwelling or piped to plot/yard | 1.741 | 1.503 | 2.018 | <0.001 | 0.841 | 0.546 | 1.298 | 0.435 |
|  | **Sanitation (referent: unimproved)** |  |  |  |  |  |  |  |  |
|  | toilet has either piped sewage system or septic tank | 1.375 | 1.188 | 1.592 | <0.001 | 0.688 | 0.439 | 1.077 | 0.102 |
|  | **Currently breastfeeding (referent: no)** |  |  |  |  |  |  |  |  |
|  | yes | 0.984 | 0.862 | 1.124 | 0.813 | 0.661 | 0.416 | 1.050 | 0.080 |
